# Conceptually mapping how investing in essential public health functions (EPHFs) and common goods for health (CGH) can improve health system performance

**DOI:** 10.3389/fpubh.2025.1531837

**Published:** 2025-09-19

**Authors:** Michelle Amri, Dheepa Rajan, Kira Koch, Alexandra J. Earle, Susan P. Sparkes, Yu Zhang, Sohel Saikat, Jesse B. Bump

**Affiliations:** ^1^The W. Maurice Young Centre for Applied Ethics, School of Population and Public Health, University of British Columbia, Vancouver, BC, Canada; ^2^European Observatory on Health Systems and Policies, WHO European Centre for Health Policy, Brussels, Belgium; ^3^Special Programme on Primary Health Care, World Health Organization, Geneva, Switzerland; ^4^Department of Health Financing and Economics, World Health Organization, Geneva, Switzerland; ^5^Takemi Program in International Health, Harvard T.H. Chan School of Public Health, Harvard University, Boston, MA, United States; ^6^Department of Global Health and Primary Care, Bergen Centre for Ethics and Priority Setting, University of Bergen, Bergen, Norway

**Keywords:** health system, good governance, health system performance assessment, essential public health functions, common goods for health, global health, health system performance, international health

## Abstract

**Background:**

Calls for investing in essential public health functions (EPHFs) and common goods for health (CGH) are numerous, but it is often unclear to policymakers how such investments lead to health system improvements.

**Objectives:**

To showcase plausible pathways between actions taken to improve specific health system functions—in other words, investments in EPHFs and CGH—and their impact on health system performance, the health systems performance assessment framework for Universal Health Coverage is used. We draw on three examples—community engagement and social participation, taxes and subsidies, and public health surveillance and monitoring—to demonstrate how action in these areas can improve health systems.

**Conclusions:**

This conceptual mapping also points to the crucial role of good governance and demonstrates how investing in multiple EPHFs and CGH can trigger a chain reaction to spur broader health system improvement.

## Introduction

Calls for governments to invest in essential public health functions (EPHFs) and common goods for health (CGH) are numerous (1–8), yet investments are often lacking. It is often unclear to policymakers how investments in EPHFs and CGH can lead to health system improvements ex-ante (9, 10). Thus, this article sketches plausible pathways to demonstrate how investing in EPHFs and CGH can improve health system performance. Our narrative elucidates the chain of events linking investments in EPHFs and CGHs to improvements in health system performance through “pathways”.

EPHFs and CGH have a similar list of key interventions and functions needed for resilient, primary health care (PHC)-focused health systems—meaning systems that empower people and communities, foster multi-sectoral policy and action, and ensure integrated service delivery. Yet, they arise from different underlying theoretical arguments. CGH are population-level, collective action areas required for public health and use economic theory about market and government failures around public goods to make the case for investment in these areas. An overview of CGH categories is provided in Box 1 and the original source for a comprehensive list can be found in (11). CGH include surveillance, legislative and regulatory systems, and environmental protection measures, across sub-national, national, and international levels (11). EPHFs are normative and include a set of fundamental, interlinked, and interdependent activities within and beyond the health sector to advance public health objectives. The list of EPHFs is provided in Box 2 of this article, but for explanatory details please see (12). EPHFs include actions relevant to national, sub-national, and local levels and those that contribute to large-scale efforts to establish CGH (12–14). For example, monitoring and surveillance activities include both individual-level detection and reporting as well as population-level synthesis and are aggregated at the global level.

Box 1 **Categories of CGH.**

1. Policy and coordination (e.g., disease control policies and strategies).

2. Regulations and legislation (e.g., environmental regulations and guidelines).

3. Taxes and subsidies (e.g., taxes on products with impacts on health to create market signals leading to behavior change).

4. Information collection, analysis, and communication (e.g., surveillance systems).

5. Population services (e.g., medical and solid waste management).

Drawn from (11).

Box 2 **List of EPHFs.**

1. Public health surveillance and monitoring.

2. Public health emergency management.

3. Public health stewardship.

4. Multisectoral planning, financing, and management for public health.

5. Health protection.

6. Disease prevention and early detection.

7. Health promotion.

8. Community engagement and social participation.

9. Public health workforce development.

10. Health service quality and equity.

11. Public health research, evaluation, and knowledge.

12. Access to and utilization of health products, supplies, equipment, and technologies.

Drawn from (13).

To showcase plausible pathways between actions taken in specific EPHFs and CGH to improve health system performance, the health systems performance assessment (HSPA) framework for Universal Health Coverage (UHC) is used. The HSPA framework not only provides a conceptual basis to orient analyses of health system data, but it also links inputs made within health system “functions” to broader health system goals (15). It is conceptual in nature and not intended to guarantee definitive outcomes, given that policy contexts will inevitably vary.

**Figure 1:**
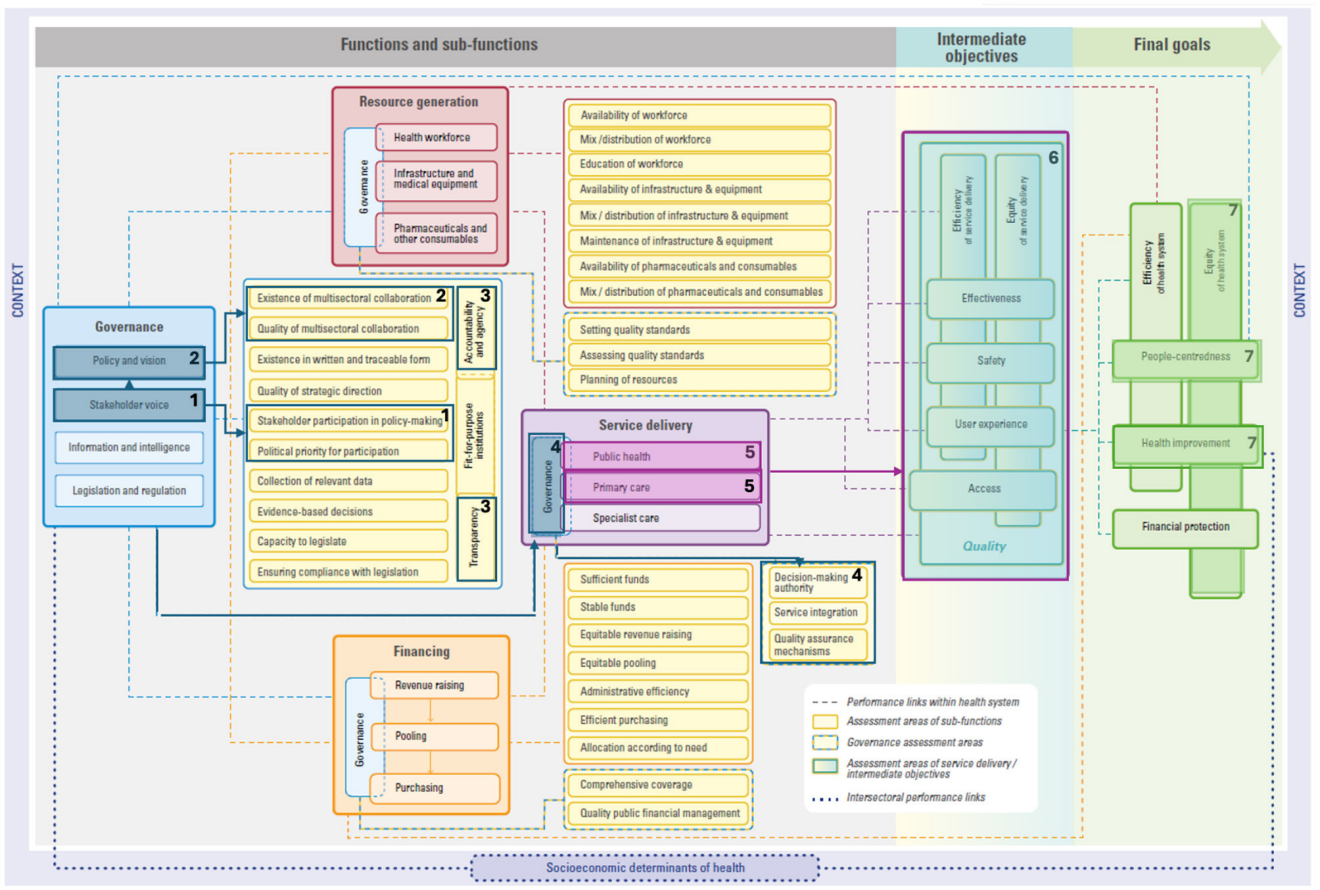
Linking community engagement and social participation to the HSPA framework. Please see (15) for the original figure.

**Figure 2:**
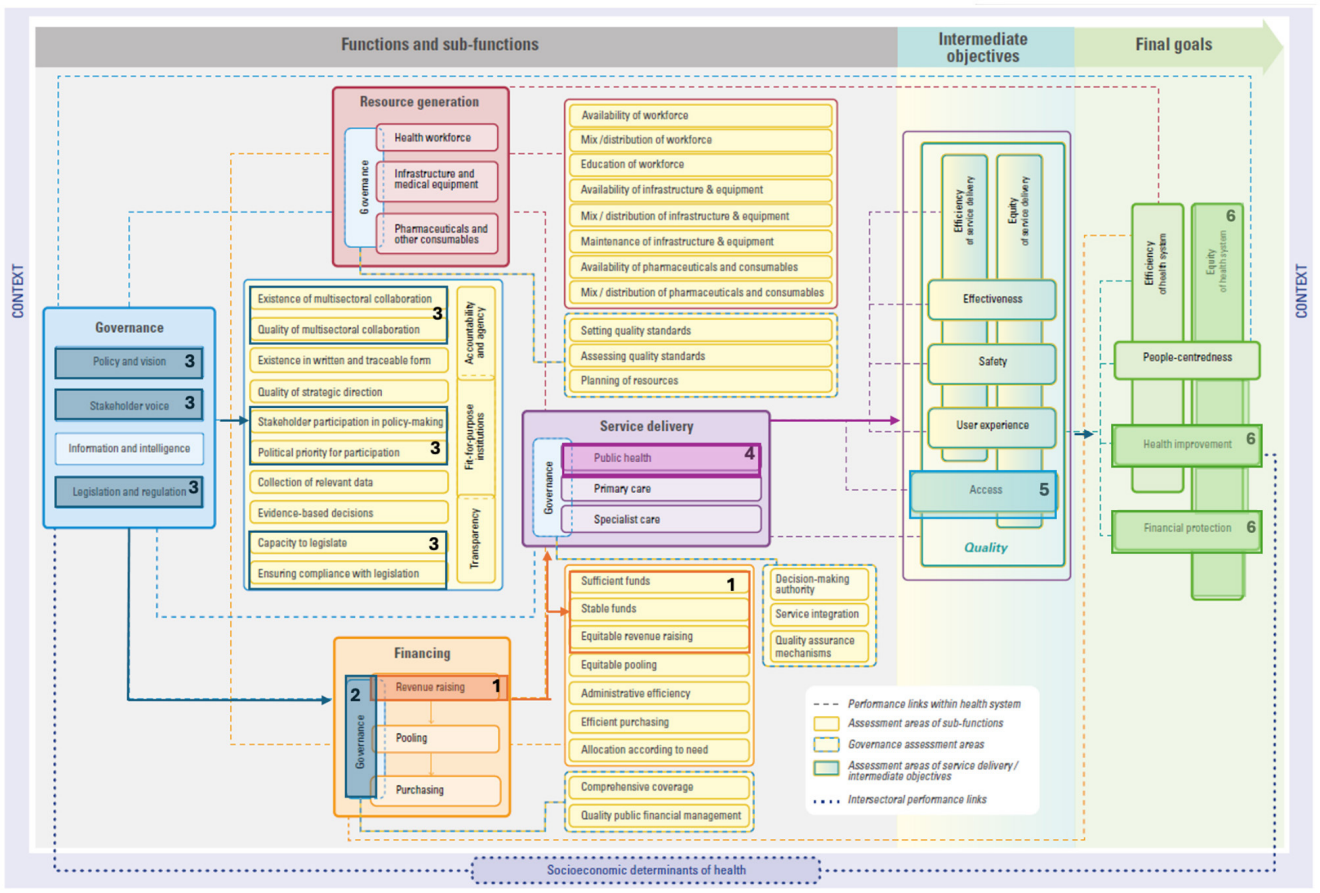
Linking taxes and subsidies to the HSPA framework. Please see (15) for the original figure.

**Figure 3:**
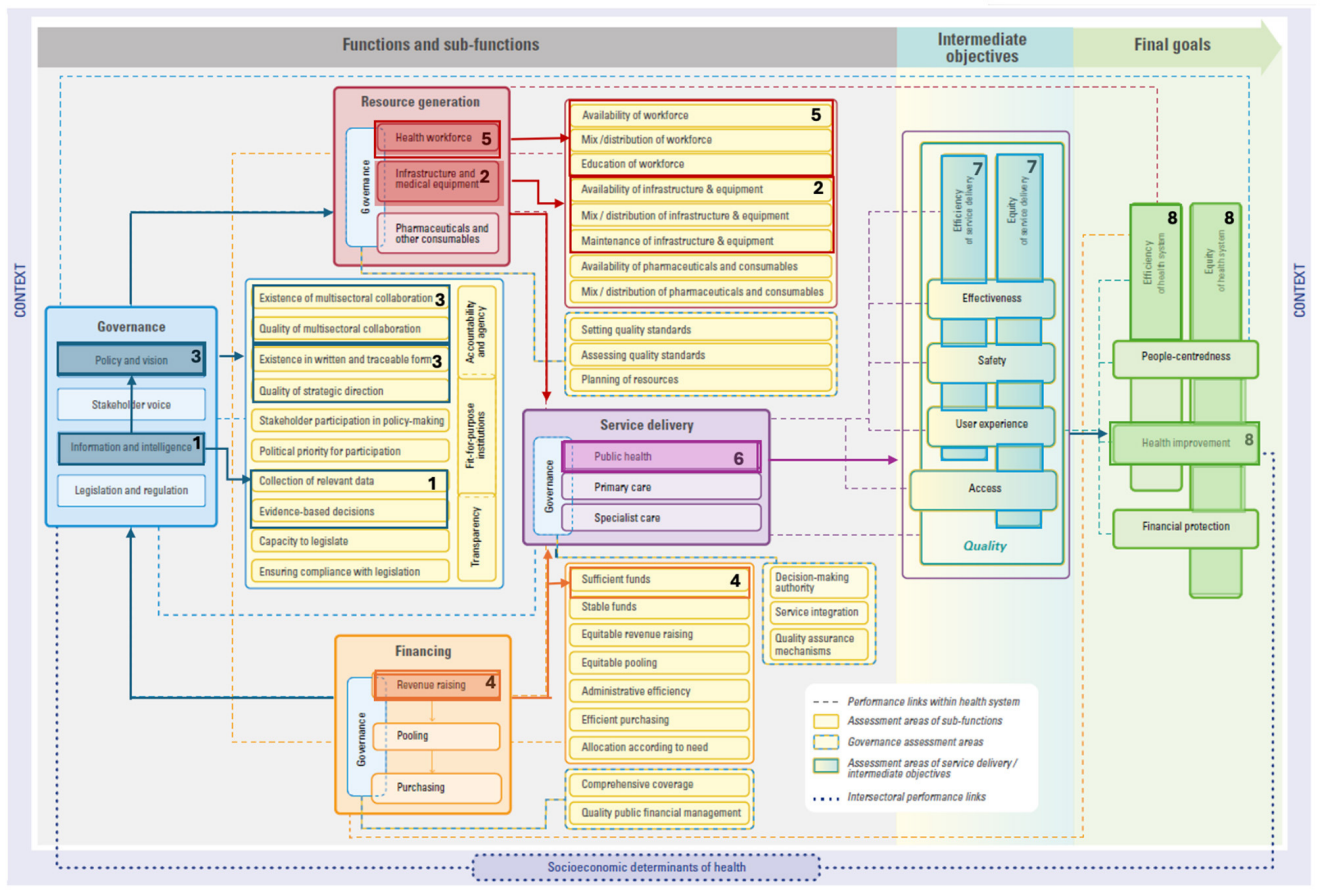
Linking public health surveillance and monitoring to the HSPA framework. Please see (15) for the original figure.

We draw on three examples embedded within both EPHF and CGH frameworks to demonstrate how investing in these areas impacts health system performance, per the HSPA framework. These examples are: (i) community engagement and social participation, (ii) taxes and subsidies, and (iii) public health surveillance and monitoring. These described links are all drawn in each respective figure to guide understandings (Figures 1–3) and details on the mechanics of the HSPA framework are provided in Box 3.

Box 3 **Mechanics of the HSPA framework.**

Within the HSPA framework, possible links between functions, sub-functions, and assessment areas are drawn with a dotted line.

*Functions* are the foundations of a health system. These functions are governance, resource generation, financing, and service delivery. Each of these functions are illustrated in the example figures (Figures 1–3), whereby governance is in a blue box, resource generation is in a red box, financing is in an orange box, and service delivery is in a purple box.

*Sub-functions* are specific actions or necessary elements that are conducive to achieving the function's objectives. In other words, sub-functions are key pieces or processes that are needed for each function. For example, within the governance function, there are four sub-functions (policy and vision, stakeholder voice, information and intelligence, and legislation and regulation). Evidently, each of these pieces are needed for ‘good' governance.

*Assessment areas* are areas that allow for examining the performance of the sub-functions. In other words, assessment areas provide the opportunity to determine how well sub-functions are performing. For example, in the case of the policy and vision sub-function of governance, assessment areas include the existence of documents in written and traceable form, quality of strategic direction, and existence and quality of multisectoral collaboration.

This conceptual mapping can be used to advance policymaker understandings of the potential pathways between EPHFs and CGH and improved health system performance. Improved understandings can help with political prioritization of and investments in EPHFs and CGH. To the best of our knowledge, this article is the first of its kind to draw conceptual links between EPHFs and CGH and health systems outcomes using the HSPA framework.

## Example 1: community engagement and social participation

*In this example, we begin with* ***community engagement and social participation****. We demonstrate that by making governance processes more* ***participatory*** *and* ***inclusive****, public health and primary care can be improved to reach the health system goals of* ***equity*** *and* ***people-centeredness****, as well as improving* ***population health***.

Our first pathway example focuses on **community engagement and social participation** (12, 16). This refers to strengthening meaningful engagement with people, communities, and civil society in strategic decision-making and service delivery (see Box 4). We start from the governance function within the HSPA framework to illustrate how investing in and prioritizing community engagement and social participation can improve health system performance, as shown in Figure 1.

Box 4 **Community engagement and social participation.**

**Community engagement and social participation** refers to strengthening community engagement, participation, and social mobilization for health and wellbeing (12). More specifically, **social participation** includes amplifying people's voices in public policy decision-making processes, whereas **community engagement** narrows in on service delivery and program design (e.g., health promotion and health literacy campaigns).

The meaningful engagement of people, communities, and civil society in decision-making processes that affect their health and wellbeing is a key sub-function of governance and labeled as **stakeholder voice** in the HSPA framework. Investing in community engagement and social participation means investing in the stakeholder voice sub-function of health system governance.

Although there is no single definition of “good governance” (17, 18), almost all governance frameworks include stakeholder participation and voice in policy development and implementation as a prominent, integral element (19–21). Ensuring an inclusive culture of participation starts with making **participation a political priority** (see #1 in Figure 1). This means not only planning for adequate resources to be devoted to social participation, but also building government capacity to design spaces for participants, particularly those with unheard voices and less power to influence debates and policy-making (**stakeholder participation in policy-making**) (22) (see #1 in Figure 1). Consequently, the stakeholder voice sub-function is closely linked to **policy and vision** (see #2 in Figure 1), to ensure policies, strategies, and plans are more responsive to population needs.

Governance that purposefully seeks to be responsive to people's needs and expectations is at the heart of **accountability**; linked to this is respecting and increasing people's **agency** for their own health (18). Accountability here refers to government accountability to the public who will be affected by policy decisions (19). A well-designed participatory process also emphasizes **transparency** in objectives, roles and mandates, and selection criteria for participants (see #3 in Figure 1). An example of this pathway segment is seen in the Tunisian Societal Dialogue for Health. The Societal Dialogue is a civil society-initiated and government-supported partnership that organized a series of large consultative meetings (23). These meetings engaged the government, civil society organizations, and citizens to not only co-design the first post-2011 revolution national health policy, but also empower individuals and communities (23, 24). Despite challenges linked to fluctuating political commitment toward participation, the Societal Dialogue has increased trust among actors and improved policy formulation by taking diverse population needs and views into account (24).

Strong governance requires the inclusion of **stakeholder voices** and clear **policy and vision**, which drives excellence across the health system. In other words, collating population views, demands, and needs to guide policies, strategies, and plans is a core aspect of better governance. Better governance has repercussions across other functions, namely financing, resource generation, and service delivery.

“Good” governance, one of the functions within the HSPA framework, enables the **service delivery** function of health systems performance. The need for good governance is demonstrated by the emphasis placed on **community empowerment** within the PHC paradigm (25). By strengthening community engagement initiatives, governance of service delivery is enhanced. For example, in Brazil, community representation in local health councils influences service integration and/or quality standards (see #4 in Figure 1) (26). The presence of a strong community voice will positively influence **public health** and **primary care** sub-functions of service delivery (see #5 in Figure 1)—both encompassed in the PHC approach (27).

**Service delivery** is assessed through indicators of **access** and **quality** of care (28). Studies demonstrate that the quality of care dimensions of **effectiveness** (29, 30), **safety** (28), and **user experience** (31–35) improve when PHC is implemented as per the principles laid out in the Astana Declaration (36), where a commitment to empowering individuals and communities is outlined (37). Additionally, many of the strategies applied to improve quality of care also aim to improve **equity** and **efficiency**. For example, efficiency may be improved through the early management of health problems (i.e., avoiding unnecessary hospitalizations), appropriate prescribing practices, and enhanced care coordination (28) (see #6 in Figure 1).

Ultimately, providing care that is effective, safe, and satisfies users (i.e., high-quality care) leads to various **health improvements**, such as reduced morbidity and mortality (28), and satisfying users can improve **people-centeredness**. And in fact, community-oriented primary health care has been found to substantially positively impact the health of underserved populations (28, 38, 39), entailing improvements in **equity** (see #7 in Figure 1).

## Example 2: taxes and subsidies

*In this second example, we begin with* ***taxes and subsidies***. *We demonstrate how taxes and subsidies can positively impact the* ***health system goals of health*** ***improvement, equity, and financial protection*** *by strengthening the* ***revenue raising*** *sub-function of financing and the* ***public health*** *sub-function of service delivery*.

**Taxes and subsidies** influence individual behavior (40) and can shape markets through the provision of positive and negative financial incentives (41). For these population-level fiscal instruments to work effectively, mechanisms to collect, pool, and distribute revenue are needed, which are generally within the purview of national governments (42).

As an illustrative example, the Government of Thailand introduced alcohol and tobacco excise taxes. By introducing these excise taxes, the **revenue raising** sub-function of **financing** is enhanced (see #1 in Figure 2), contributing to sufficient and stable funds as part of overall government spending devoted to health.

For these fiscal instruments to work effectively, **governance of health financing** arrangements must be established, including public financial management (see #2 in Figure 2). Additionally, making decisions regarding the introduction of health taxes requires a strong **governance function** throughout the process, particularly in areas related to **policy and vision**, **multisectoral collaboration** between ministries of health and finance alongside other stakeholders, as well as **legislation and regulation** (see #3 in Figure 2).

Excise taxes can also create market signals for **health promotion** (43)—a core aspect of the **public health** sub-function of service delivery (see #4 in Figure 2). In Thailand, the excise tax was used to create ThaiHealth, a health promotion agency with a broad mandate. ThaiHealth's activities encompass addressing noncommunicable diseases, health protection, disease prevention, and early detection (44, 45).

The funds raised through these taxes are part of overall tax revenues that form the basis of public financing for UHC. These funds can lead to more and better-quality public health services which improves **access**, an intermediate HSPA framework objective (see #5 in Figure 2), and **financial protection**, a HSPA framework final goal (see #7 in Figure 2). In other words, improving access to quality health services based on need and not ability to pay, which is a core tenant of UHC. As stressed in the World Health Report 2010, public spending is needed to effectively expand coverage and move toward UHC (46). This requires an adequate tax base, which can come from a range of instruments, including health taxes and subsidies. Ultimately, by improving **financial protection**, we can expect **improvements to health** given that user fees largely deter health system use (47), which is another HSPA framework final goal (see #6 in Figure 2). Additionally, **equity** in the health system may be improved given that most health financing mechanisms have an equity aspect (48), which is an overarching HSPA framework final goal (see #6 in Figure 2). Similarly, a greater influx of taxes and subsidies can support more **equitable** health financing by improving **financial protection** against the risk of ill health [i.e., ensuring access to services without financial hardship (49)]. This is especially important given that the COVID-19 pandemic has afforded recognition of the problem of heightened inequity (50) and resulted in those paying out of pocket being more likely to experience worsened financial hardship (51).

Levying health taxes and ensuring equitable health financing moves a health system closer to not only the system goal of **equity**—recognizing that many approaches to health equity exist (52–55)—but also **financial protection**. In the case of ThaiHealth that targets noncommunicable diseases, there are ties to financial protection, as socioeconomic differences are linked to differing outcomes (56).

## Example 3: public health surveillance and monitoring

*In this final example, we delve into the links between* ***public health surveillance and*** ***monitoring*** *and the health systems goals of* ***access*** *and* ***health improvement****. In drawing this link, we explain how public health surveillance and monitoring, such as through investing in data governance, infrastructure, and interoperable digital platforms, is crucial for both governance and resource generation*.

Investing in **public health surveillance and monitoring** involves strengthening capacities of health authorities to collect, research, analyze, monitor, and evaluate data on a regular basis and understand how to use that evidence to undertake informed decisions (57). Prioritizing this area requires investing in data governance, represented in the **information and intelligence** sub-function of the health system governance function (see #1 in Figure 3). Further, investing in public health surveillance and monitoring also requires data infrastructure to support interoperability of digital platforms. Infrastructure needs to be supported at the community level (e.g., within primary care) to the national level. Such efforts are represented in the **infrastructure and equipment** sub-function of resource generation (see #2 in Figure 3).

A strong information and intelligence sub-function is closely linked with the **policy and vision** sub-function (see #3 in Figure 3). Evidently, timely and disaggregated data on population health status, risk, protective and promotive factors, threats to health and health system performance, and service utilization can guide policymakers to understand causes of poor health, track progress, and adjust decision-making and implementation strategies (58, 59). Further, disease surveillance and response requires strong leadership and strategic direction to address gaps (e.g., cold chain challenges) and ensure an effective response. The importance of governance is particularly clear when considering that epidemics can proliferate in the absence of sufficient responses, as was the case for the Ebola virus disease outbreak in western Africa in 2014 when it was declared a public health emergency of international concern (60). However, since then, numerous actions have been taken around public health surveillance and monitoring. In the case of Liberia, although the integrated disease surveillance and response strategy was adopted in 2004, it did not allow for an effective response to the Ebola epidemic from 2014 to 2016 due to not securing resources and an abundance of vertical programs (61). Ultimately, after Liberia actively implemented the strategy in 2015 (62), this led to successes and lessons learned around the importance of strong governance, namely around **multisectoral collaboration** (see #3 in Figure 3), partnership, local ownership, and promoting the PHC approach (63). Moreover, disease surveillance and monitoring also requires the development and implementation of coordination platforms and systems, as well as sectoral and sub-sectoral policies and strategies.

**Disease surveillance and monitoring** requires adequate resources to support continued efforts and the implementation of response measures. In fact, non-sustainable financial resources was identified across 33 studies as a main issue in implementing the Integrated Disease Surveillance and Response System, an approach that uses one system to collect data about multiple diseases or behaviors (64). Funds need to be allocated for collecting, analyzing, and disseminating data (65). Additionally, disease surveillance and monitoring requires enough trained and competent health workers to carry out the necessary activities, as inadequately trained staff and staff turnover can pose a challenge for disease surveillance (64). Evidently, these activities are linked to **financing** (e.g., **revenue raising**, see #4 in Figure 3) and **resource generation** (e.g., **health workforce**, see #5 in Figure 3). Ultimately, undertaking public health surveillance and monitoring requires **governance**, **financing**, **resource generation**, and **service delivery**, particularly in public health (see #6 in Figure 3). Through a more targeted public health surveillance approach, outreach activities can improve **equity** by better reaching at risk populations while generating **efficiency** gains to avoid disease spread (see #7 in Figure 3). Thus, improving **equity**, **efficiency**, and **health** within the health system (see #8 in Figure 3). An example of this pathway is demonstrated in Lesotho. When handling the COVID-19 pandemic, rising comorbidities and excess mortality was observed as resulting from both communicable and noncommunicable diseases (66). The government was able to use this data to make the evidence-based decision to scale-up interventions to better target susceptible populations and improve health security (66).

## Conclusion

Drawing a direct pathway from one action taken to a final health system objective is difficult. However, via three examples—community engagement and social participation, taxes and subsidies, and public health surveillance and monitoring—we demonstrate how actions in these areas can improve health system performance. By exploring these links, the chain of events to improved health system performance is traced. Our intention is not to provide a prescriptive formula for bringing about particular changes but rather to offer a conceptual mapping that visualizes connections between actions taken and broader impacts on health system goals. Future analyses can also explore drawing conceptual links across additional frameworks or concepts employed by other major institutions (e.g., the European Commission, the Organization for Economic Co-operation and Development, and the World Bank). Additionally, future investigations can measure how specific precursors influence outputs and outcomes, including potential unintended consequences and how they can be mitigated.

Notably, this conceptual mapping also points to the crucial nature of “good governance” which appeared in all three examples. Per the World Bank, good governance “is synonymous with sound development management” (67). Although there is no single definition, different sources outline various key principles. One set of principles include openness, participation, accountability, effectiveness, and coherence (68). Another identifies strategic vision, participation and consensus orientation, rule of law, transparency, responsiveness, equity and inclusiveness, effectiveness and efficiency, and ethics (69). And another, equity, participation, organizational arrangements, accountability, integrity, and transparency (70). Good governance is particularly essential due to the very nature of EPHFs and CGH, as both involve addressing collective action failures among health and non-health stakeholders. Governance anchored in inclusive decision-making with strong coordination across stakeholders is key and can improve financing, resource generation, and service delivery functions to lead to various performance objectives. In fact, participation is thought to be a necessary condition for the other four principles in the first set of good governance principles mentioned above (71).

Although we assessed how specific actions impact health system performance, it is not enough to act in only one area. Investing in multiple EPHFs and CGH can trigger a chain reaction to bring about broader system change. To achieve health system goals, various EPHFs and CGH should be invested in, strengthened, and politically prioritized. Policymakers can not only use our conceptual mapping to improve understandings of interlinkages between frameworks, but to negotiate for increased investments.
